# Risk Perception and Knowledge Following a Social Game–Based Tobacco Prevention Program for Adolescents: Pilot Randomized Comparative Trial

**DOI:** 10.2196/63296

**Published:** 2024-11-05

**Authors:** Georges Khalil, Erica Ramirez, Meerah Khan, Bairu Zhao, Nuno Ribeiro, Patrick Balian

**Affiliations:** 1 Department of Health Outcomes and Biomedical Informatics University of Florida Gainesville, FL United States; 2 Cancer Prevention Unit Institute of Pathology and Molecular Immunology of University of Porto and Institute for Research and Innovation in Health University of Porto Porto Portugal; 3 Warrington College of Business University of Florida Gainesville, FL United States

**Keywords:** tobacco prevention, vaping, combustible tobacco, risk perception, adolescent, games, social interaction

## Abstract

**Background:**

Adolescence is a critical developmental stage that is particularly vulnerable to the initiation of tobacco use. Despite the well-documented health risks associated with tobacco use, it remains prevalent among adolescents. Games for health are a promising strategy for tobacco prevention, using experiential and social learning theories to enhance engagement and improve behavior change.

**Objective:**

This pilot study aims to (1) compare the social game–based program Storm-Heroes to a nonsocial program regarding adolescents’ personal and social experiences and (2) examine how these experiences predict higher tobacco knowledge and perceived risks of vaping and conventional tobacco use.

**Methods:**

In a cluster-randomized comparative design, 4 after-school sites (N=79 adolescents) were recruited in person and randomized in a single-blinded format to 1 of 2 interventions: the social game Storm-Heroes (44/79, 56%) or the nonsocial program A Smoking Prevention Interactive Experience (ASPIRE; 35/79, 44%). A study team member supervised both interventions. Data were collected at baseline, immediate follow-up, and a 1.5-month follow-up (45/74, 61% retained). Repeated measures mixed effects models were conducted.

**Results:**

A total of 45 participants continued until the 1.5-month follow-up. Participants in the Strom-Heroes group were more likely to increase their perceived risk of vaping (B=0.40; *P*<.001), perceived risk of conventional tobacco use (B=0.35; *P*=.046), and tobacco knowledge (B=1.63; *P*<.001) than those in the control condition. The usability level of the program was related to a higher perceived risk of vaping (B=0.16; *P*=.003) and conventional tobacco use (B=0.16; *P*=.02) by follow-up. Attention to the program was also related to higher perceived risk of vaping (B=0.12; *P*=.002) and conventional tobacco use (B=0.14; *P*<.001). Distraction was not related to either perceived risk of vaping (*P*=.15) or perceived risk of conventional tobacco use (*P*=.71). In contrast, both more attention (B=0.60; *P*<.001) and less distraction (B=–0.37; *P*<.001) were related to higher tobacco knowledge.

**Conclusions:**

The increased perceived risk of vaping and conventional tobacco among Storm-Heroes participants aligns with the program’s goals of improving participants’ awareness of the risks associated with tobacco use and their tobacco knowledge. However, distraction weakened the effect of the program on tobacco knowledge, indicating that emphasis needs to be placed on minimizing distraction for better outcomes. With the results of this study, researchers can work to advance the current version of Storm-Heroes and amplify engagement in the program to improve its potential for preventing adolescents’ initiation of tobacco use.

**Trial Registration:**

ClinicalTrials.gov NCT02703597; https://clinicaltrials.gov/study/NCT02703597

## Introduction

### Background

Adolescence is a critical developmental stage that is particularly vulnerable to the initiation of tobacco use [1-3]. Research indicates that exposure to nicotine during this period is associated with substantial impairments in brain growth, psychological harm, and long-term physical health outcomes [4,5]. Despite the well-known risks associated with adolescent tobacco use, the rates of use among this age group remain a concern. In 2023, approximately 28% of high-school students and 14.7% of middle-school students reported ever using a tobacco product [6,7]. Of the youth who reported ever using tobacco products, approximately 50% of them are current tobacco users [6,7]. As a result, the need for tobacco prevention is evident.

One particularly promising strategy for tobacco prevention is the application of games for health. Gameplay can include an amalgam of entertainment and education strategies to drive health behavior change [8]. On the basis of the experiential learning theory and the social learning theory, the immersive nature of gameplay facilitates a successful learning process through a playful and entertaining environment [9,10]. Game-based interventions can increase motivation, engagement, and overall sustainability of health behaviors [11]. In addition to our work, researchers have shown the success of games for health through randomized controlled trials [12-15].

Engaging gameplay has proven to be a promising avenue for tobacco prevention [16]. On the basis of a systematic review [17], most games for combustible tobacco prevention and cessation have leveraged the use of rewards and interactive activities to drive behavior change. Among these interventions, success was primarily observed in smoking cessation games rather than in prevention efforts [17,18]. In contrast, games meant for vaping or e-cigarette prevention have recently shown success. One example is a game called “Invite Only VR,” which showed improvement in e-cigarette knowledge, nicotine addiction knowledge, perceived addictiveness of e-cigarettes, and perceptions of harm [19]. In addition, 1 comprehensive game covering vaping and combustible tobacco, smokeSCREEN, improved antitobacco beliefs and tobacco knowledge [20]. These results highlight the potential success of game-based interventions.

By including various gaming elements (eg, competition, collaboration, reward, goal setting, and storytelling), games can provide flexibility in addressing different issues pertaining to tobacco use. One qualitative study for the design of tobacco prevention games examined adolescents’ gaming preferences and showcased the elements of cooperation, storytelling, and physical performance as key experiential learning elements for tobacco prevention [21]. The findings suggest that gaming elements can be combined to design an effective and engaging tool that covers the complexities of different tobacco products and addresses unique topics pertaining to this risky behavior.

### A Social Game–Based Intervention

This line of research on gaming elements for tobacco prevention led to the design of a social game–based intervention, called Storm-Heroes, which is ideal for education systems (eg, schools and after-school programs). As a social game, Storm-Heroes offers adolescents the opportunity to witness and model healthy behaviors, such as rejecting tobacco, thereby promoting tobacco risk education [21]. With social gaming, Storm-Heroes relies on the social learning theory by promoting interpersonal discussions and boosting self-efficacy through the practice of skills to stay tobacco free. In addition, Storm-Heroes conveys normative feedback, influencing adolescents’ risk perceptions regarding tobacco use [21]. This aligns with the health beliefs model, which posits that psychosocial factors such as social interaction and peer pressure can promote risk perception and ultimately encourage behavior change [22]. Through these mechanisms, Storm-Heroes serves as a tool for tobacco prevention, leveraging peer influence and normative feedback to positively impact adolescents’ perceptions of tobacco use risks and improve knowledge.

### Study Objectives

The purpose of this pilot study is to (1) compare the social game–based program Storm-Heroes to a nonsocial program with respect to adolescents’ personal and social experience with the program and (2) examine the role of adolescents’ experience with the program in predicting higher perceived risk of vaping, perceived risk of conventional tobacco use, and knowledge by follow-up. Table 1 clarifies the hypotheses tested in this study.

**Table 1:** List of hypotheses.

Types of hypothesis	Hypothesis statements
Personal experience	Engagement with Storm-Heroes will result in higher attention, lower distraction, and higher recognition of program imagery than engagement with a nonsocial equivalent program. Engagement with Storm-Heroes will result in perceptions of better usability, higher level of fun, better narrative quality, more enjoyment, and more creative freedom than engagement with a nonsocial equivalent program.
Social experience	Engagement with Storm-Heroes will result in higher engagement in peer-to-peer discussions and better quality of discussions than engagement with a nonsocial equivalent program.
Tobacco-related outcomes	Engagement with Storm-Heroes will result in improved perceived risk of vaping and higher perceived risk of conventional tobacco use (cigarettes, cigars, and little cigars) than engagement with a nonsocial equivalent program. Engagement with Storm-Heroes will result in improved tobacco knowledge than engagement with a nonsocial equivalent program.
User experience mechanisms of change	Personal experience factors and social interactivity will predict higher perceived risk of vaping, perceived risk of conventional tobacco use, and tobacco knowledge by follow-up while controlling for program allocation.

## Methods

### Study Design

To pilot-test adolescents’ experience with Storm-Heroes, this study involved a 2-arm single-blinded cluster-randomized comparative trial. The pilot study was conducted in June 2021 at 4 after-school sites in Florida, and it was registered at the ClinicalTrials.gov registry, as part of a larger study (identifier: NCT02703597). Its components adhere to the CONSORT (Consolidated Standards of Reporting Trials) and CONSORT-EHEALTH (Consolidated Standards of Reporting Trials of Electronic and Mobile Health Applications and Online Telehealth) guidelines [23,24]. We assessed demographic information at baseline and program experience at immediate follow-up. We measured perceived risk of vaping, perceived risk of combustible tobacco use, and tobacco knowledge at baseline and 1.5-month follow-up.

### A Brief Description of the Interventions

We compared Storm-Heroes with A Smoking Prevention Interactive Experience (ASPIRE), a nonsocial program that is similar to Storm-Heroes in terms of session structure and type of health content. Table 2 describes the differences and similarities in design elements for Storm-Heroes and ASPIRE. A detailed description of the intervention is provided in Multimedia Appendix 1 [25-32], which follows the Template for Intervention Description and Replication (TIDieR) checklist [33]. Multimedia Appendix 2 presents the CONSORT-EHEALTH items.

**Table 2:** Differences and similarities in elements for Storm-Heroes and A Smoking Prevention Interactive Experience (ASPIRE).

Elements	Storm-Heroes	ASPIRE	Mechanism of action
Health content based on TTM^a^	Yes	Yes	Presenting both programs with the TTM ensures that adolescents receive consistent health information and persuasive messaging based on the processes of change.
Entertainment-education videos	Yes	Yes	By leveraging narratives, these videos deliver messages that correct misconceptions about the prevalence and acceptability of tobacco use, shaping adolescents’ perceptions to align with healthy norms.
Human-computer interaction	Yes	Yes	This interaction engages adolescents through a digital platform, making learning about tobacco risks engaging and interactive.
Gameplay	Yes	No	Gameplay improves engagement and provides a simulated environment where adolescents can learn through experience and build confidence.
Social interaction	Yes	No	Social interaction encourages adolescents to discuss tobacco risks and refusal skills with peers, enhancing their understanding and commitment to staying tobacco free.
Hybrid format	Yes	No	Combines online and offline activities to keep adolescents engaged and reinforce learning in various contexts.
Dosage and frequency	5 weekly sessions for 45 min each	5 weekly sessions for 45 min each	The dosage and frequency ensure that adolescents receive similar and consistent exposure for both programs.

^a^TTM: transtheoretical model.

ASPIRE is a computer-guided intervention that uses engaging videos and interactive activities across 5 sessions over 5 weeks, with each session lasting approximately 45 minutes. It aims to enhance information retention and guide adolescents toward a tobacco-free lifestyle by engaging users through text, animations, videos, and activities. ASPIRE is evidence based and tested for tobacco prevention. The intervention program is freely accessible over the web [34,35].

**Figure 1:**
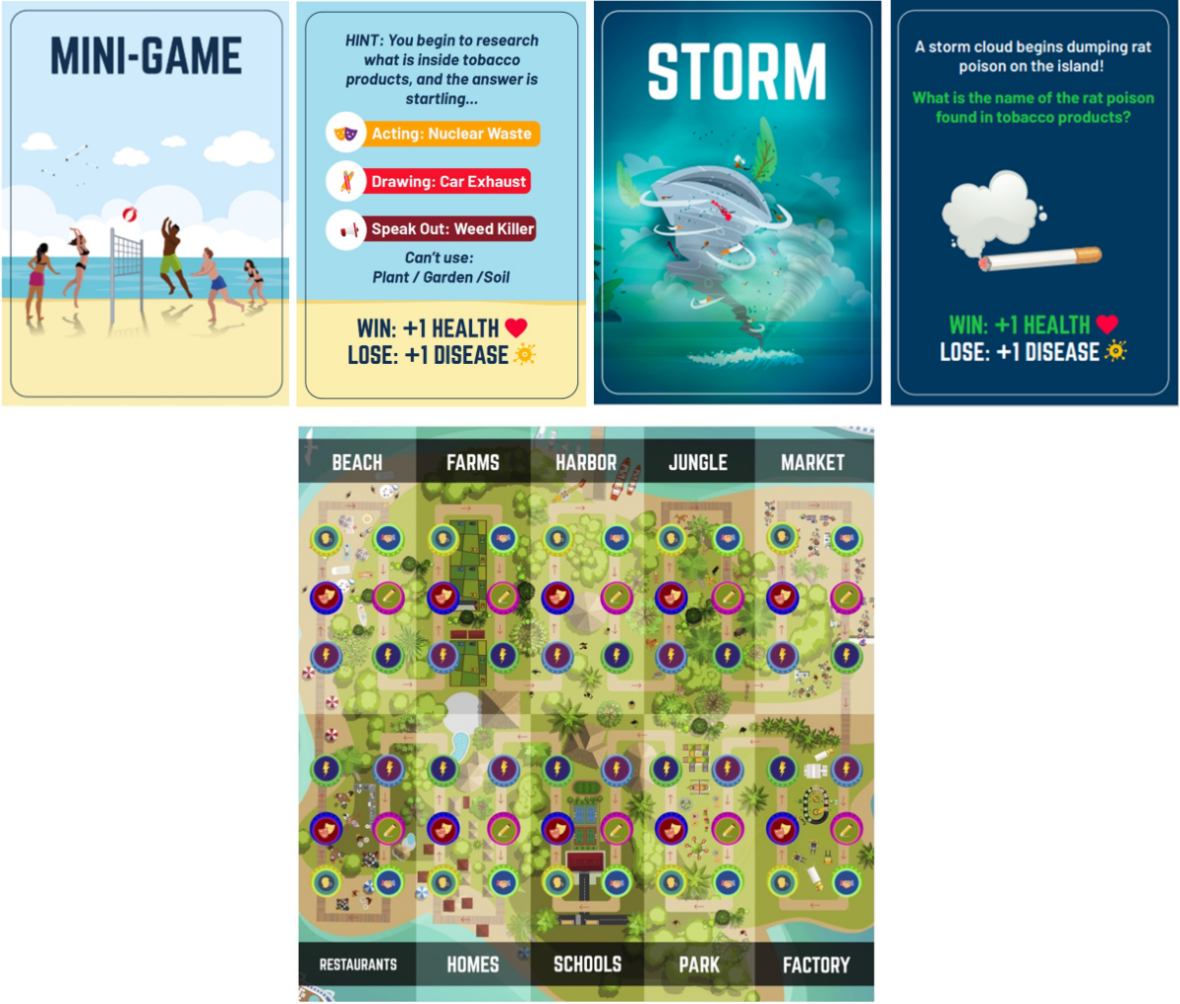
Depiction of Storm-Heroes activities and game board.

The Storm-Heroes intervention was developed collaboratively, involving a game designer, a research team with tobacco education expertise, and a youth design committee. Messages were designed using scientific evidence and message-framing strategies to impact tobacco risk perception, knowledge, and intention to use. The intervention aims to educate adolescents about tobacco risks, environmental consequences, and impacts on social and mental well-being, incorporating 367 unique antitobacco messages based on the transtheoretical model [36] and empowerment theory. The design process resulted in a dynamic and socially engaging educational program, Storm-Heroes, combining digital and in-person elements. It seamlessly integrates web-based components with game-based tabletop activities, including ASPIRE-derived videos and game-based social activities for group interaction (Figure 1).

In Storm-Heroes, adolescents engage within a narrative. They play the role of friends on an island struck by a storm bringing tobacco products, harmful chemicals, and disease. To combat the storm’s effects and save their island, teams embark on quests, participating in entertainment-education videos and activities. Before engaging in the program, adolescents are grouped using a validated social network algorithm. The grouping process ensures that each participant with high intentions to use tobacco is grouped with close friends who do not intend to use tobacco, facilitating constructive support during activities. Storm-Heroes offers adolescents 5 main activities delivered on validated board game material. These include trivia with multiple-choice questions, acting where one member silently acts as others guess, drawing for guessing from sketches, speaking out for verbal clues, and teamwork scenarios presenting group dilemmas. The activities aim to engage teams in collaborative problem-solving around tobacco-related topics. Multimedia Appendix 1 describes the activities and how they are presented to players. The materials of Storm-Heroes include informative background information in game-based social activities, such as scripts and task instructions, a tabletop game board, decks for board game cards, dice, tokens, and pons. The materials can be accessed by reaching out to the researchers.

Both ASPIRE and Storm-Heroes cover a comprehensive list of health topics related to tobacco, including its composition, effects on the body and brain, environmental impact, and strategies for tobacco prevention and advocacy (Multimedia Appendix 1). The content is structured consistently across both programs, covering aspects from understanding tobacco to building skills for a tobacco-free lifestyle and community activism.

### Ethical Considerations

The institutional review board for human subject research at the University of Florida approved this study (IRB201903082). Adolescents and their parents were informed of the study’s purpose and procedure. They provided written informed parental consent and written informed child assent. During data collection sessions, participants were reminded of the study purpose, procedures, risks, and that they could withdraw from the study at any time. Participants completed the surveys in a private classroom with supervision. Participants were asked to maintain the confidentiality of their own identity and the identity of other participants. Participants received up to US $50 as compensation. Data were deidentified before analysis.

### Study Procedures

A total of 4 after-school sites located in Florida were randomly selected for recruitment. After approval from the program directors, a verbal announcement reached adolescents at these sites, and interested adolescents completed child assent and parental permission. For participation, adolescents needed to be aged 11 to 18 years (inclusive) and enrolled in a middle school or high school. Adolescents also needed to be comfortable using a computer and the internet.

During recruitment, adolescents and their parents were informed offline that the study aimed to improve adolescent health through an interactive program in Florida after-school sites; that the study may take approximately 2 months and 1 week; and that they will engage in activities, surveys, and interviews. The incentive was described to potential participants, and they were informed that participation is voluntary and confidential and that the data would be securely stored at the University of Florida. Recruitment and data collection took 3 months to complete.

Participants started their experience with the intervention 3 to 7 days after they completed the baseline survey. The statistical team generated the random allocation of sites to each condition. In ASPIRE and Storm-Heroes, participants used similar computers and had private classrooms for participation. A study team member was available for technical assistance and supervision. A volunteer site staff trained in youth engagement was present to ensure that participants did not deviate from the requested data collection procedures. Participants completed surveys in a supervised classroom setting immediately after the intervention and again 1.5 months later. Participants completed other survey assessments at follow-up (data not included in this paper).

### Implementation of Each Intervention

Our study staff were trained to implement the program at the after-school sites. They traveled to each study site location to administer the ASPIRE and the Storm-Heroes programs. Participants were not told which intervention was of interest to the researchers. During the site visits, study staff recorded attendance, ensured that the appropriate regimen was implemented, and addressed any questions or concerns participants had during the sessions.

At each site designated to receive ASPIRE, adolescents engaged in five 1-hour sessions exclusively focused on the full ASPIRE program. This regimen was conducted similarly to previous work on ASPIRE. At each site designated to receive Storm-Heroes, participants were first organized into groups comprising 3 to 6 individuals, determined by the outcomes of the social network survey conducted at baseline. With the aid of the social network algorithm, the study team grouped each at-risk adolescent (those indicating the highest intention to use tobacco) with 2 to 5 of their closest peers exhibiting lower intention to use tobacco. Unexpectedly, it was observed that some participants were absent during certain sessions. As a result, the grouping was re-evaluated using the algorithm for the sessions when participants were absent. Within their groups, participants were instructed to engage in ASPIRE activities, followed by game-based social activities within the board game. The duration of board game play varied for each session depending on the length of the assigned ASPIRE activity.

### Measures

We assessed the measures through web-based closed surveys in a classroom setting, and a study staff was available for assistance. Survey measures have been previously tested and validated. Multimedia Appendix 3 [34,37-47] includes a detailed description of the main measures, measure references, and Cronbach α values when applicable.

At baseline only, we included survey questions pertaining to potential confounders and demographic characteristics, including age, sex at birth, ethnicity, race, average grade at school, the number of detentions at school, parents’ highest level of education, and perceived skills in playing board games. At baseline, we also measured the status of using vaping products, cigarettes, and cigars or little cigars using the Minnesota smoking index [37].

At both baseline and 1.5-month follow-up, we measured perceived risk of vaping, perceived risk of using conventional tobacco products (cigarettes, cigars, and little cigars), and tobacco knowledge [38,39] (Multimedia Appendix 3).

At immediate follow-up, we collected data regarding participants’ experience with each of the programs using validated measures. First, to check for expected differences and similarities between the ASPIRE and the Storm-Heroes conditions, we assessed measures pertaining to key program features. We expected group differences with respect to perceived social interactivity [40] and group similarities with respect to attitude toward the program, visual esthetics, and emotional involvement [41-44]. Next, to assess engagement, we measured recognition of program imagery, attention to the program, and distraction from the program [45-47]. To capture user experience, we assessed participants’ perceptions regarding the usability of the program [42,43], level of fun [41], narrative quality [42,43], program enjoyment [34], and creative freedom [42,43]. Considering the role of social interaction in the success of Storm-Heroes, we asked participants to indicate if they engaged in any discussions with their peers after the program. If they confirmed that they engaged in discussions, they were then asked to share the content of their discussion through an open-ended qualitative question. With a mixed methods approach, the qualitative responses were analyzed and coded to identify if participants discussed the program or tobacco (coded 1) or not (coded 0).

### Statistical Analysis

We conducted statistical analyses using Stata (version 14; StataCorp LP). Considering cluster-randomization, we used multilevel generalized linear mixed effects models (GLMMs). For all GLMMs, we identified demographic characteristics that may need to be included in the models. In every GLMM, an after-school site was modeled as a random effect nested within the intervention condition, and the intervention condition and time (and their interaction) were modeled as fixed effects. GLMMs use maximum likelihood estimation, producing unbiased estimates when data are assumed to be not missing completely at random.

To conduct GLMMs using a target power of 0.85 and an effect size of 0.23 to perceived risk of vaping with an α value of .05, the estimated sample size was 45 participants [39]. We estimated that 75 adolescents would be needed to test the hypotheses, with an anticipated completion rate of approximately 60% (45/75). Considering the pilot nature of this study, this sample size was considered sufficient for the study of short-term secondary outcomes.

First, with GLMMs, we tested for any baseline differences between the 2 conditions with respect to demographic characteristics (eg, age, sex at birth, gender identity, grades at school, number of detentions, parental education level, and perceived skills playing board games). Second, with one-way ANOVA and chi-square tests, we examined attrition by testing differences between those retained and those lost to follow-up with respect to the outcome variables at baseline and other potential confounding factors.

Next, with GLMMs, we examined group differences for outcomes of interest. We conducted GLMMs predicting 5 types of outcomes. Manipulation check outcomes included perceived social interactivity, attitude toward the program, visual esthetics, and emotional involvement. Outcomes pertaining to participants’ attention to the program included general attention, distraction from the program, and recognition of imagery from the program. Personal experience with the program included perceptions regarding program usability, level of fun, narrative quality in the program, program enjoyment, and creative freedom. Communication outcomes included engagement in discussions and quality of discussions. Tobacco-related outcomes included perceived risk of vaping, perceived risk of conventional tobacco use, and tobacco knowledge. Models predicting tobacco-related outcomes included group assignment, time, and the group-by-time interaction term as predictors. Following these models, we examined the role of attention to the program and personal experience factors in predicting tobacco-related outcomes.

For qualitative data, we conducted a thematic analysis of participants’ responses to the open-ended question on engagement in discussion. We aimed to look for themes pertaining to tobacco, the program, or both. Next, we generated a binary variable that indicates if participants positively discussed tobacco or the program.

## Results

### Participants

In terms of demographics, the average age of the participants was 13.55 (SD 1.65) years, with 5% (40/74) of the participants aged ≤13 years, 58% (43/74) being female at birth, and most (56/72, 78%) being Black or African American. Approximately 37% (26/70) of the participants reported having at least 1 friend who vapes, and approximately 13% (9/69) of the participants reported having at least 1 friend who smokes a combustible product. Table 3 presents the demographic characteristics by group.

**Table 3:** Baseline participants’ characteristics.

Characteristics			Total sample (N=74)		Storm-Heroes (n=39)		ASPIRE^a^ (n=35)		*P* value	
**Age (y), n (%)**										.96^b^
	≤13	40 (56)		19 (49)		21 (64)				
	>13	32 (44)		20 (51)		12 (36)				
**Sex at birth, n (%)**										.83^b^
	Male	31 (42)		21 (54)		10 (29)				
	Female	43 (58)		18 (46)		25 (71)				
**Race** **, n (%)**										.17^b^
	Black or African American	56 (78)		35 (90)		21 (64)				
	Not Black or African American	16 (22)		4 (10)		12 (36)				
**Ethnicity, n (%)**										.96^b^
	Hispanic or Latinx	16 (22)		6 (15)		10 (30)				
	Not Hispanic or Latinx	56 (78)		33 (85)		23 (69)				
**Grades at school, n (%)**										.31^b^
	Mostly A	35 (48)		16 (41)		19 (56)				
	Mostly B or C	38 (52)		23 (59)		15 (44)				
**Parents’ level of education, n (%)**										.50^b^
	Received a college degree	53 (73)		27 (69)		26 (76)				
	Did not receive a college degree	20 (27)		12 (31)		8 (23)				
**Number of detentions at school, n (%)**										.45^b^
	None	58 (78)		26 (67)		32 (91)				
	≥1	10 (13)		9 (23)		1 (3)				
	≥2	6 (8)		4 (10)		2 (6)				
Perceived board game skills, mean (SD)			3.45 (0.91)		3.49 (0.84)		3.41 (0.99)		.82^c^	
Number of friends who vape, mean (SD)			3.14 (12.32)		1.29 (2.83)		5.34 (17.86)		.11^c^	
Number of friends who smoke, mean (SD)			0.68 (2.54)		0.55 (2.390)		0.84 (2.76)		.52^c^	
Perceived risk of vaping, mean (SD)			3.15 (0.95)		3.07 (0.97)		3.25 (0.93)		.49^c^	
Perceived risk of conventional tobacco use, mean (SD)			3.31 (0.82)		3.26 (0.81)		3.37 (0.84)		.41^c^	
Tobacco knowledge, mean (SD)			10.30 (3.07)		10.45 (3.19)		10.14 (2.99)		.58^c^	

^a^ASPIRE: A Smoking Prevention Interactive Experience.

^b^Significance testing with mixed effect logistic regression to adjust for group randomization (categorical variables).

^c^Significance testing with mixed effect regression to adjust for group randomization (continuous variables).

### Attrition

**Figure 2:**
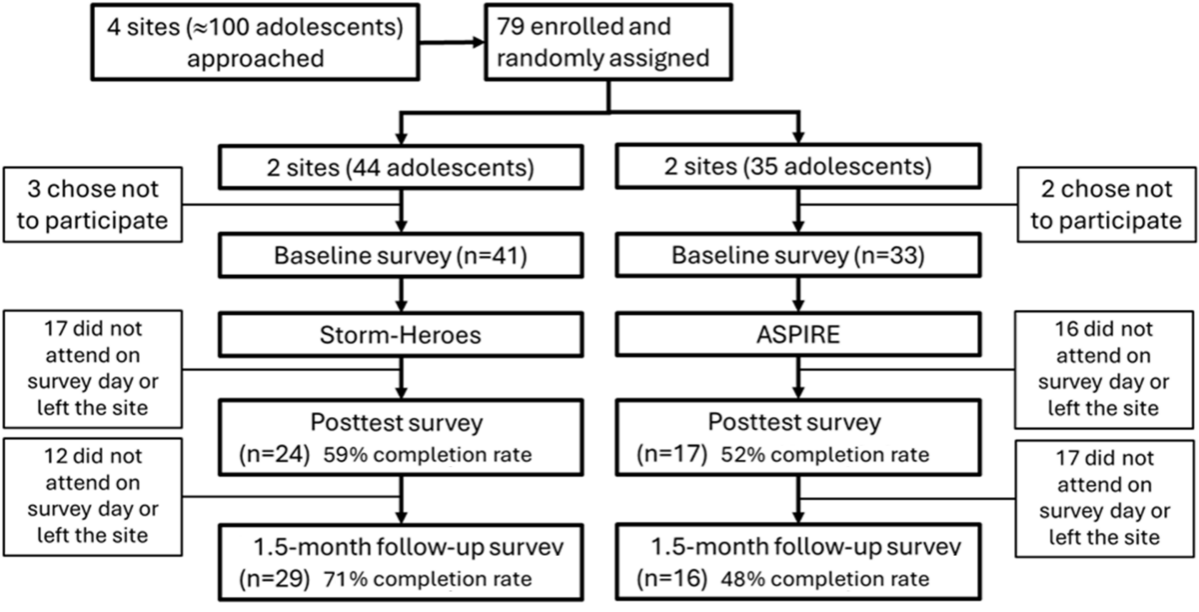
CONSORT (Consolidated Standards of Reporting Trials) flow diagram. Participants were allowed to participate in any survey assessment over time. ASPIRE: A Smoking Prevention Interactive Experience.

No harm or unintended effects occurred in this study. In this study, >100 adolescents expressed interest, and 79 enrolled, with the 4 sites randomly assigned to either Storm-Heroes or ASPIRE. In total, 94% (74/79) of the adolescents completed the baseline survey. Among baseline participants, 55% (41/74) participated in the posttest experience survey and 61% (45/74) participated in the 1.5-month follow-up survey (Figure 2). Participants who did not complete surveys had left the after-school site or did not attend the site on the day of data collection.

Participants in Storm-Heroes were as likely to continue to follow-up assessment as those in ASPIRE (χ^2^_1_=3.2; *P*=.07). There were no significant differences between participants who did and those who did not continue to the 1.5-month follow-up with respect to baseline perceived risk of vaping (*F*_1,73_=3.74; *P*=.06), perceived risk of conventional tobacco use (*F*_1,73_=2.43; *P*=.12), tobacco knowledge (*F*_1,58_=2.40; *P*=.13), sex at birth (χ^2^_1_=0.5; *P*=.50), age (*F*_1,76_=0.94; *P*=.34), being Black or African American (χ^2^_1_=2.2; *P*=.14), being Hispanic or Latinx (χ^2^_1_=0.1; *P*=.75), grades at school (χ^2^_1_=0.6; *P*=.44), parents’ level of education (χ^2^_1_=0.2; *P*=.34), the number of friends who vape (*F*_1,68_=0.59; *P*=.44), the number of friends who smoke (*F*_1,67_=2.92; *P*=.09), or perceived skills in playing board games (*F*_1,70_=0.57; *P*=.45). These data are further detailed in Multimedia Appendix 4.

### Manipulation Checks

We checked to ensure that participants expressed positive attitudes toward both interventions equally. There was no significant difference between the 2 conditions with respect to attitude scores (B=0.76; *P*=.07) or visual esthetics of the program (B=1.28; *P*=.14). With both interventions being entertainment based, there was no significant difference between the conditions with respect to being emotionally involved in the content (B=0.31; *P*=.56). As expected, participants who received Storm-Heroes perceived the program to be more socially interactive than those who received ASPIRE (B=5.33; *P*=.02).

### Checking for Confounders

We tested potential confounding effects of demographic characteristics. There were no significant differential effects on perceived risk of vaping as a function of perceived board game skills, race, or the number of detentions. Being younger (*P*<.001), being male (*P*=.001), being non-Hispanic (*P*=.02), having friends who vape (*P*=.001), having friends who smoke (*P*<.001), and having parents with lower education level (*P*=.04) moderated the effect of Storm-Heroes on perceived risk of vaping.

There were no significant differential effects on perceived risk of conventional tobacco use as a function of age, perceived board game skills, ethnicity, race, and number of detentions. Being male (*P*<.001), having friends who vape (*P*<.001), having friends who smoke (*P*<.001), and having parents with lower education level (*P*=.04) moderated the effect of Storm-Heroes on perceived risk of conventional tobacco use.

There were no significant differential effects on tobacco knowledge as a function of age, ethnicity, race, or the number of detentions. Having lower boardgame skills (*P*<.001), being female (*P*=.001), being non-Hispanic (*P*<.001), having friends who vape (*P*<.001), having friends who smoke (*P*=.01), and having parents with lower education level (*P*<.001) moderated the effect of Storm-Heroes on tobacco knowledge.

### Personal Experience

Mixed effects models controlling for confounders showed that participants who received Storm-Heroes were significantly more likely to be distracted during the program (B=1.36; *P*=.002) and less likely to recognize images from the program (B=1.68; *P*<.001). However, they were more likely to pay attention to the program than those who received ASPIRE (B=1.30; *P*=.02). By examining the interaction between intervention groups and distraction, we found that distraction weakened the effect of Storm-Heroes on recognition of program imagery (B=–0.49; *P*=.005).

Participants who received Storm-Heroes found the program to have significantly better usability (B=0.88; *P*=.001), higher level of fun (B=4.14; *P*=.001), better narrative quality (B=2.66; *P*=.001), more enjoyment (B=2.16; *P*=.047), and more creative freedom (B=1.90; *P*=.047) than participants who received ASPIRE.

### Communication Outcomes

Participants who received Storm-Heroes were significantly more likely to talk to others during the program (odds ratio [OR] 4.99, 95% CI 1.04-23.85; *P*=.04). They also experienced a better quality of peer-to-peer discussions (B=2.16; *P*=.047). According to the open-ended questions about the content of their discussions, participants who received Storm-Heroes were significantly more likely to discuss the program and the negative effects of tobacco with their peers than those who received ASPIRE (OR 5.63, 95% CI 1.25-25.29; *P*=.02). By examining the role of social interactivity, it was found that participants who found the program to be socially interactive were approximately twice as likely to talk about the program and the negative effects of tobacco (OR 1.98, 95% CI 1.28-3.07; *P*=.002).

### Tobacco-Related Outcomes

**Figure 3:**
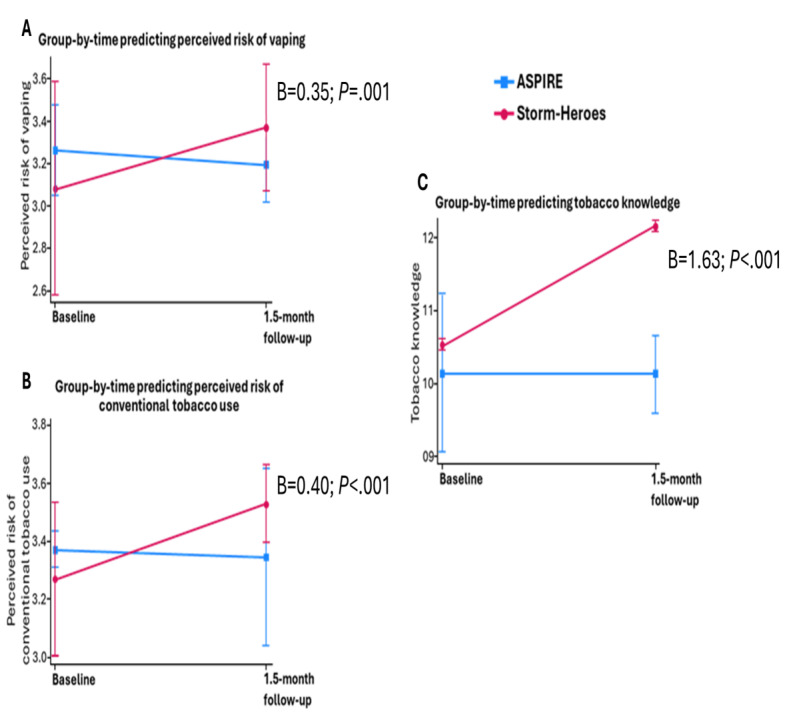
Adjusted predictions of condition-by-time. Coefficients and *P* values show significance of the group-by-time interaction effect. Perceived risk measures can range between 1 and 4, while tobacco knowledge can range between 0 and 22. ASPIRE: A Smoking Prevention Interactive Experience.

Mixed effects models indicated that group allocation by time was significantly related to perceived risk of vaping (group-by-time: B=0.35; *P*=.001; Figure 3A). Participants who received Storm-Heroes were significantly more likely to exhibit a higher perceived risk of vaping at follow-up than participants who received ASPIRE, controlling for perceived risk of vaping at baseline (B=0.40; *P*=.02; Table 4).

**Table 4:** Multilevel models predicting the perceived risk and tobacco knowledge.

	Model 1: predicting the perceived risk of vaping at follow-up (n=42)		Model 2: predicting the perceived risk of conventional tobacco use at follow-up (n=42)		Model 3: predicting tobacco knowledge at follow-up (n=19)	
	B (SE)	*P* value	B (SE)	*P* value	B (SE)	*P* value
Receiving Storm-Heroes	0.40 (0.17)	.02	0.35 (0.18)	.046	1.75 (0.56)	.002
Perceived risk of vaping at baseline	0.60 (0.17)	<.001	—^a^	—	—	—
Perceived risk of conventional tobacco use at baseline	—	—	0.68 (0.19)	<.001	—	—
Tobacco knowledge at baseline	—	—	—	—	0.53 (0.21)	.01
Number of detentions	–0.44 (0.31)	.16	–0.28 (0.15)	.06	—	—
Average grades at school	—	—	—	—	0.93 (0.21)	<.001
Parents’ level of education	—	—	—	—	0.76 (1.84)	.68

^a^Not available; the models were fitted based on identified key covariates.

Participants in the Storm-Heroes condition were significantly more likely to exhibit an increase in perceived risk of conventional tobacco than participants in the ASPIRE condition (group-by-time: B=0.40; *P*<.001; Figure 3B). Participants in the Storm-Heroes condition were significantly more likely to exhibit higher perceived risk of conventional tobacco use at follow-up than participants in the ASPIRE condition, controlling for perceived risk of conventional tobacco at baseline (B=0.35; *P*=.046; Table 4).

Participants in the Storm-Heroes condition were significantly more likely to exhibit an increase in tobacco knowledge than participants in the ASPIRE condition (group-by-time: B=1.63; *P*<.001; Figure 3C). Participants in the Storm-Heroes condition were significantly more likely to exhibit higher tobacco knowledge at follow-up than participants in the ASPIRE condition, controlling for tobacco knowledge at baseline (B=0.53; *P*=.01; Table 4).

### Experience Factors Predicting Tobacco-Related Outcomes

Controlling for group allocation, the results showed that the usability level of the program was related to a higher perceived risk of vaping (B=0.16; *P*=.003) and conventional tobacco use (B=0.16; *P*=.02) by follow-up. Attention to the program was also related to higher perceived risk of vaping (B=0.12; *P*=.002) and conventional tobacco (B=0.14; *P*<.001). Distraction was not related to either perceived risk of vaping (*P*=.15) or perceived risk of conventional tobacco use (*P*=.71). In contrast, both more attention (B=0.60; *P*<.001) and less distraction (B=–0.37; *P*<.001) were related to higher tobacco knowledge. A follow-up exploratory analysis of moderation indicated that distraction weakened the effect of receiving Storm-Heroes on tobacco knowledge by follow-up (group-by-distraction: B=–6.67; *P*<.001).

## Discussion

### Principal Findings and Comparison to Prior Work

This paper describes a pilot cluster-randomized comparative trial examining the short-term effectiveness of Storm-Heroes, a social game–based intervention, in improving secondary tobacco-related outcomes, including perceived risk of tobacco use and tobacco knowledge. The paper also presents results from adolescents’ experience with the intervention and its prediction of such outcomes. We hypothesized that adolescents’ engagement with Storm-Heroes would result in (1) better quality of program experience; (2) improved perceived risk of vaping and conventional tobacco use; and (3) improved tobacco knowledge compared with the engagement in ASPIRE, a nonsocial, non–game-based equivalent program.

The increased perceived risk of vaping and conventional tobacco among Storm-Heroes participants aligns with the program’s goals of improving participants’ awareness of the risks associated with tobacco use. With antitobacco messages designed to communicate tobacco risk, Storm-Heroes may have effectively presented the severity of tobacco-related harm. The comprehensive content in Storm-Heroes is designed with key risk communication strategies, including emotionally involving gain-framed and loss-framed messages that cover the psychological, physiological, medical, and environmental consequences of tobacco use [25]. In addition, along with other theoretical frameworks, the program design is grounded in the health belief model and empowerment theory, promoting perceived susceptibility [48] and self-efficacy [49,50]. The game-based social activities in Storm-Heroes allow adolescents to engage in interactive learning experiences that empower them to recognize and internalize the harms of tobacco, motivating them toward tobacco-free lifestyles [21].

The Storm-Heroes group showed a significant increase in tobacco knowledge scores from baseline to 1.5-month follow-up. Knowledge gained among Storm-Heroes participants compared to ASPIRE participants may be the outcome of both exposure to information within the program and increased motivation to seek information elsewhere (eg, from school teachers, the internet, etc). First, by integrating multimedia resources and a proactive learning approach, Storm-Heroes aimed to equip adolescents with comprehensive knowledge. As supported by previous research [25], the program’s tobacco education content was carefully designed to cover several key topics (Multimedia Appendix 1) and promote a holistic understanding of information from a wide array of tobacco products [25]. In addition, the gameplay aspect of Storm-Heroes encourages structured information retention that can support knowledge gain. Second, gameplay and other entertainment-based programming have been shown to stimulate interest in understanding health issues and ultimately promote health information seeking beyond the content of a program [12,51,52]. This information-seeking behavior may ultimately contribute to increased knowledge. In addition to the tobacco-related outcomes, we identified user experience differences between the 2 programs.

In the context of program experience, our results indicate that participants expressed similar positive attitudes toward both programs, with no significant difference in attitude scores or perceived visual esthetics. Emotional involvement in the content was also similar between the 2 groups. However, Storm-Heroes was perceived as more socially interactive than ASPIRE. Supportive of previous research, the similar attitude toward both interventions suggests that entertainment-based approaches, regardless of social interactivity, can effectively engage adolescents. However, Storm-Heroes was perceived as more socially interactive than ASPIRE, which aligns with previous findings indicating that interactive elements enhance program appeal [21,53,54]. As supported by the social learning theory and a systematic review of tobacco education programs, incorporating social features into interventions can promote health behavior change by fostering a sense of peer support for adolescents [55]. Our results suggest that while entertainment-based approaches effectively engage adolescents, perceived social interactivity of Storm-Heroes may play a unique role in its success.

Our results further indicated that participants in the Storm-Heroes program were more likely to engage in conversations with others during the program and experienced better-quality peer-to-peer discussions compared to those in the ASPIRE program. They were more likely to discuss the program and the harm of tobacco use. This suggests that Storm-Heroes may have included strategies that successfully encourage healthy dialogues among participants. Theoretical frameworks such as the extended elaboration likelihood model [56] support the ability of entertainment-based programming to promote healthy interpersonal discussions. This has been particularly evident when it comes to sensitive health topics such as contraceptive use, organ donation, and underage tobacco use [52,57-59]. Our results show that participants who found the program to be socially interactive were more likely to engage in healthy discussions. In line with the social learning theory, social interaction can facilitate social modeling and promote healthy learning [60,61]. Future research could further investigate the mechanisms that allow Storm-Heroes to drive these communication outcomes.

Participants who received Storm-Heroes were more likely to be distracted during the program and less likely to recall images from it. Nevertheless, they were more attentive overall compared to ASPIRE recipients. While Storm-Heroes led to more distractions and lower image recall, its higher attention levels imply deeper engagement despite potential distractions. However, the challenge lies in balancing engagement with lower distractions, as distractions may undermine the program’s effectiveness. While distraction did not significantly impact perceived risk of vaping or conventional tobacco use, it was negatively associated with tobacco knowledge, thereby hindering adolescents’ learning and retention of information. Future research should focus on implementation strategies to minimize distraction for Storm-Heroes. For example, using a flipped classroom approach can allow adolescents to receive tobacco-related information at home through entertaining videos and engage in social activities in class to practice what they learned [62]. This implementation strategy can reduce cognitive load, thereby optimizing engagement without compromising health education [63].

The findings support the original mechanisms of action outlined in Table 2. The significant increase in attention among participants who received Storm-Heroes highlights the effectiveness of using engaging and interactive elements such as gameplay and social interaction. Despite an increase in distraction and a lower recognition of program imagery, participants who received Storm-Heroes reported higher levels of usability, fun, narrative quality, enjoyment, and creative freedom compared to those who received ASPIRE. These factors likely contributed to enhanced perceived risk and knowledge, as suggested by the mechanisms of action. The enjoyment and narrative quality could have facilitated social interactions and discussions about tobacco, while the creative freedom and program interactivity bolstered participants’ engagement and practice of tobacco-free skills. Although distraction diminished the program’s impact on recognizing program imagery, the overall roles of positive reception and attention underscore the potential of Storm-Heroes to effectively leverage the social learning theory and the health belief model to promote tobacco prevention among adolescents. Considering the pilot nature of this study, we invite researchers in games for health to further explore these mechanisms.

This study advances our understanding of how game-based approaches that leverage social elements can be strategically applied to address adolescent tobacco use. While existing research has highlighted the potential role of personal engagement in games in driving health outcomes [20,64], this study distinguishes itself by focusing on social gameplay. The study specifically examines the comparative effectiveness of a socially interactive game versus a nonsocial program. By integrating social interactivity with tobacco prevention strategies, the research provides new insights into how interactive elements can enhance engagement and improve outcomes such as tobacco knowledge and perceived risks.

Unlike previous studies [26,65], which have broadly addressed game-based learning, our study delves into the unique role of multiplayer gameplay and its impact on adolescent tobacco prevention. The findings underscore the unique role of social games in fostering meaningful peer-to-peer discussions and elevating tobacco risk awareness among adolescents. Moreover, while previous research highlighted the need to boost engagement by lowering the negative effect of social influence [26,65], this study demonstrates that multiplayer gameplay can be affected by distractions, and minimizing these distractions can optimize the educational impact of social games.

### Limitations

First, this study ended with a relatively low retention rate (45/74, 61%). By the time this study reached 1.5-month follow-up, adolescents were at a transition out of the after-school summer period, entering the fall semester, and ultimately, several of them were not available to continue in the study. In addition, several participants had to leave early during the data collection at the 1.5-month follow-up, which led to a low sample of participants who completed the knowledge index. This low retention rate could have influenced the study’s results by reducing the generalizability and statistical power. While our use of repeated measures mixed effects modeling allowed us to account for missing data, future studies could mitigate this limitation by planning data collection at more stable periods, such as during school class sessions, and by enrolling a larger initial sample size to account for potential dropouts.

Second, although this study showed a change in short-term outcomes (ie, tobacco risk perception and knowledge), we did not examine a long-term change in tobacco use behavior. It must be noted, though, that this early pilot trial was meant to test the potential for adolescents’ experience with Strom-Heroes to drive risk perception and knowledge. The lack of long-term behavioral data may limit the ability to draw conclusions about the program’s effectiveness in reducing actual tobacco use over time. To address this, future research should include longer follow-up periods and assess behavioral outcomes to provide a more comprehensive evaluation of the program’s impact.

Third, this study did not examine the specific types of discussions adolescents engaged in during their interactions. Without a detailed analysis of the content of these discussions, it is not possible to investigate how the program influences communication behaviors or the outcomes of these discussions. Future studies could enhance the research by incorporating qualitative methods, such as focus groups or interviews, to explore the content and context of these discussions, thereby providing deeper insights into the program’s impact on social interactions.

Fourth, from an implementation perspective, the study required staff members to deliver the program to each classroom and moderate the sessions. This approach may limit the program’s scalability and dissemination potential. The reliance on staff for program delivery could also introduce variability in implementation quality. To improve this, future efforts should consider adapting the procedures to allow teachers to deliver the program. This would not only facilitate broader reach but also create a more sustainable implementation model. Using Proctor’s Framework for Implementation Outcomes, future studies could evaluate the program by assessing teachers’ adherence to key steps, the quality of their engagement, their satisfaction, and the perceived feasibility of the program.

### Implications

The results of this study suggest that Storm-Heroes can be a promising intervention for tobacco prevention. Nevertheless, we must further examine strategies that may allow us to minimize distractions while maximizing engagement to boost the success of this intervention. Once the design of this program is clear, it becomes possible to further investigate its success by examining its long-term effects on actual tobacco use. In addition, promoting peer-to-peer interactions can improve the impact of such interventions by facilitating knowledge dissemination and perceived tobacco risks. In the long run, going beyond these short-term outcomes, randomized trials with longitudinal data collection can provide valuable insights into the success of Storm-Heroes in preventing actual initiation of tobacco use and identify the factors that may promote long-term prevention outcomes. Future researchers can work to identify the specific program components and delivery methods that contribute to enhancing adolescents’ experience and improving tobacco-related outcomes. In addition, by identifying effective components responsible for an improved program experience, we can design novel interventions that can be tailored to target specific groups of adolescents and address their unique needs concerning different tobacco products.

## Appendix

Multimedia Appendix 1 A description of the interventions based on the Template for Intervention Description and Replication (TIDieR) checklist.

Multimedia Appendix 2 CONSORT-EHEALTH (Consolidated Standards of Reporting Trials of Electronic and Mobile Health Applications and Online Telehealth) checklist.

Multimedia Appendix 3 Main study measures.

Multimedia Appendix 4 Participant characteristics based on attrition.

## Abbreviations

ASPIRE: A Smoking Prevention Interactive Experience
CONSORT: Consolidated Standards of Reporting Trials
CONSORT-EHEALTH: Consolidated Standards of Reporting Trials of Electronic and Mobile Health Applications and Online Telehealth
GLMM: generalized linear mixed effects model
OR: odds ratio
TIDieR: Template for Intervention Description and Replication

## Data Availability

The datasets generated and analyzed during this study are not publicly available due to security purposes requested by the academic institution but are available from the corresponding author on reasonable request.
