# Supplementary material for: Risk Perception and Knowledge Following a Social Game–Based Tobacco Prevention Program for Adolescents: Pilot Randomized Comparative Trial
Source: JMIR Serious Games. 2024 Nov 5;12:e63296. doi: 10.2196/63296 (PMC11576604; doi:10.2196/63296)
Supplement: Multimedia Appendix 1 [file games_v12i1e63296_app1.docx]

**Multimedia Appendix 1: A Description of the Interventions Based on The Template for Intervention Description and Replication (TIDieR) Checklist**


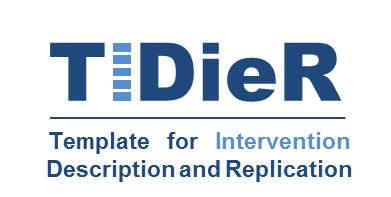


**The TIDieR (Template for Intervention Description and Replication) Checklist*:**

Information to include when describing an intervention and the location of the information

| **Item number** | **Item** | **Where located** | |
| --- | --- | --- | --- |
|  |  | Primary paper  (section title) | Location in Appendix 1 |
|  | **BRIEF NAME** |  |  |
| **1.** | *Provide the name or a phrase that describes the intervention.*  The name of the intervention is Storm-Heroes. | “Introduction”, under “A Social-Game-based Intervention | Page x |
|  | **WHY** |  |  |
| **2.** | *Describe any rationale, theory, or goal of the elements essential to the intervention*.  Storm Heroes utilized experiential learning theory, social learning theory, and the transtheoretical model which have been shown to be effective in behavioral change interventions. Storm Heroes also incorporated entertainment-education videos, game-based social activities, social network grouping, as well as game mechanics and health content into the intervention design. | __Introduction___ | Page x |
|  | **WHAT** |  |  |
| **3.** | *Materials: Describe any physical or informational materials used in the intervention, including those provided to participants or used in intervention delivery or in training of intervention providers. Provide information on where the materials can be accessed (e.g. online appendix, URL).*  Storm Heroes contains animations and videos as informational materials. The intervention also includes informative background information in game-based social activities, such as scripts and task instructions, a tabletop game board, decks for board game cards, dice, tokens, and pons. The materials can be accessed by reaching out to the researchers. | _Pages 3 & 4__ | Appendix 1: page x |
| **4.** | *Procedures: Describe each of the procedures, activities, and/or processes used in the intervention, including any enabling or support activities.*  A baseline assessment was used to collect social network data for adolescent grouping. The participants were then engaged in groups for five 45-minute sessions for 5 weeks. During each session, participants first engaged in ASPIRE activities for 5 minutes, and then played the board game for 10 minutes. Participants repeated this process until the session is complete. The ASPIRE group, completed their one-hour session individually using computers in private classrooms. | __Page 6____ | Appendix 1: page x |
|  | **WHO PROVIDED** |  |  |
| **5.** | *For each category of intervention provider (e.g. psychologist, nursing assistant), describe their expertise, background and any specific training given.*  In the case of this study, the intervention was delivered by the study staff (research coordinators and research assistants), who were trained to follow the intervention protocol. Their background was in public health, education, and communication. | _Page 7____ | Appendix 1: page x |
|  | **HOW** |  |  |
| **6.** | *Describe the modes of delivery (e.g. face-to-face or by some other mechanism, such as internet or telephone) of the intervention and whether it was provided individually or in a group.*  The Storm Heroes intervention was delivered face-to-face and online, the ASPIRE was delivered online only, via computers. Both interventions were delivered under face-to-face supervision at the after-school programs. | _Pages 6 & 7__ | Appendix 1: page x |
|  | **WHERE** |  |  |
| **7.** | *Describe the type(s) of location(s) where the intervention occurred, including any necessary infrastructure or relevant features.*  The intervention was implemented in various Boys and Girls Clubs locations in Northeast Florida. | __Page 6___ | Appendix 1: page x |
|  | **WHEN and HOW MUCH** |  |  |
| **8.** | *Describe the number of times the intervention was delivered and over what period of time including the number of sessions, their schedule, and their duration, intensity or dose.*  The Intervention was delivered 5 times during a 5-7 week period. The length of each session is 45-60 minutes, with interactive animation or videos and game-based social activities. | _Page 6____ | Appendix 1: page x |
|  | **TAILORING** |  |  |
| **9.** | *If the intervention was planned to be personalized, titrated, or adapted, then describe what, why, when, and how.*  Adolescent grouping in this program was personalized based on adolescents’ tobacco use risk and classroom friendships. Adolescents were grouped in the classroom using a validated social network algorithm, such that each adolescent with a high intention to use tobacco was grouped with close friends who did not intend to use tobacco. The algorithm allows at-risk adolescents to receive constructive support from close peers who have no intention of using tobacco while participating in the activities. | __________ | Appendix 1: page x |
|  | **MODIFICATIONS** |  |  |
| **10.** | *If the intervention was modified during the course of the study, describe the changes (what, why, when, and how).*  N/A | __N/A____ | _____________ |
|  | **HOW WELL** |  |  |
| **11.** | *Planned: If intervention adherence or fidelity was assessed, describe how and by whom, and if any strategies were used to maintain or improve fidelity, describe them.*  N/A | ____N/A______ | _____________ |
| **12.^ǂ^** | *Actual: If intervention adherence or fidelity was assessed, describe the extent to which the intervention was delivered as planned.*  N/A | ____N/A______ | _____________ |

**Description of Storm-Heroes, Version of Summer 2021:**

**Intervention Design.** Instead of solely relying on adolescents' input and theory, we developed the program mechanics and its health education content through a participatory design approach, with a game designer, a research team with tobacco education expertise, and a youth design committee that was actively involved in the design process and health message framing. Based on the scientific evidence from previous research, the messages in Storm-Heroes were designed to apply message-framing strategies that have been shown to impact tobacco risk perception, knowledge, and tobacco use intention for both vaping and conventional products. The design is meant to educate adolescents about the risks of tobacco use, the environmental consequences associated with it, and the impact on social and mental well-being. We developed a total of 367 unique anti-tobacco messages, that covered ten processes of change from the transtheoretical model (TTM) as well as reinforcement of beliefs and advocacy engagement.^1^ TTM is a common model of application for tobacco prevention and cessation, and its application has redundantly shown success.^2^ Essential elements of this theory were integrated to shape messages addressing both nicotine vaping and traditional tobacco use. Additionally, we applied the empowerment theory, particularly within messages that communicate about vaping.

**A Hybrid Program.** The design process resulted in a dynamic and socially engaging educational program that utilizes a hybrid approach that involves both digital and in-person components. The program seamlessly integrates an online component with a game-based tabletop segment. In particular, Storm-Heroes includes entertainment-education videos derived from entertainment-education videos and game-based social activities that allow adolescents to interact with health information and each other in groups. In the current implementation strategy, adolescents alternate between videos and activities. Storm Heroes includes informative background information in game-based social activities, such as scripts and task instructions, a tabletop game board, decks for boardgame cards, dice, tokens, and pons. The materials can be accessed by reaching out to the researchers.

**Game Narrative.** In Storm-Heroes, health content is presented within the context of an engaging narrative. Through cooperation, adolescents play the role of a group of friends living on an island. A storm hits the island, bringing with it a flurry of dark magic, spreading tobacco products, disease-causing chemicals, and nicotine. The storm causes friends, family, and fellow islanders to get sick, and it harms the environment. To save the island from the storm’s damage, each team goes on a quest and engages in entertainment-education videos and the activities.

**Adolescent Grouping.** For social interaction, adolescents are grouped in the classroom using a validated social network algorithm, such that each adolescent with high intention to use tobacco is grouped with close friends who do not intend to use tobacco.^3,4^ The algorithm allows at-risk adolescents to receive constructive support from close peers who have no intention of using tobacco while participating in the activities.

**The Entertainment-education videos.** These videos are derived from ASPIRE, a computer-guided intervention.^5^ The curriculum spans five sessions delivered over five weeks, each lasting about 45 minutes. Through human-computer interaction and without social interaction, ASPIRE aims to enhance information retention, guiding adolescents toward adopting a tobacco-free lifestyle.

**The Game-Based Social Activities.** In Storm-Heroes, adolescents are presented with a series of activities that have been designed based on participatory design research.^6^ The activities consist of five main mini-games. The activities are delivered on validated board game material and it includes five main activities. Trivia activities include multiple-choice questions about tobacco and healthy lifestyles. Teams guess the answers together. Acting activities involve one team member silently acting out the answer while others guess. Drawing activities involve a team member who sketches the answer for the team to guess. Speaking out activities involve a team member who describes the answer verbally for the team to guess. Teamwork scenarios include multiple-choice questions that present team dilemmas to solve together. Figure 1 presents an example of how some activities are presented to players.

**Health Content:**

Both ASPIRE and Storm-Heroes address the following health content:^5^ “What is tobacco?”, “What is in tobacco?”, “Tobacco and your body”, “Tobacco and the brain”, “Tobacco and your health”, “Second-hand smoke and vapor”, “Thirdhand smoke and vapor”, “Environmental consequences”, “How others see me when I use tobacco”, “Is tobacco really what I want?”, “Commit to a tobacco-free life”, “Skills to manage tobacco triggers”, “Why spread the word?”, “Skills to spread the word”, “Advocacy in the community”, and “Activism in the community”. ASPIRE covers the same list of health content, and in the same order.

**Theoretical Foundations:**

The health content of the program is built based on the transtheoretical model of behavior change, particularly through the application of the processes of change. The design of the game mechanics in Storm-Heroes is first based on the experiential learning theory (ELT).^7^ Gameplay encourages curiosity and experimentation, allowing adolescents to discover health information through exploration. In addition, based on the social learning theory (SLT), we expect that as adolescents engage in the game’s content and activities and interact with their peers, they will begin to understand the norms against tobacco use and build self-efficacy by engaging with their peers in tobacco-related information that clarifies the risks of tobacco use and the means to avoid tobacco use.^8^

**Implementation of Each Intervention:**

In the current trial, our study staff members were trained to implement the program at the after-school sites. They traveled to each study site location to administer the programs. Participants were not told which intervention was of interest to the researchers. During site visits, study staff recorded attendance, ensured the appropriate regimen was implemented, and addressed questions or concerns from participants during the sessions.

At each site designated to receive ASPIRE, adolescents assigned to receive ASPIRE engaged in five one-hour sessions exclusively focused on the full ASPIRE program. This regimen was conducted similarly to previous work on ASPIRE.

At each site designated to receive Storm-Heroes, participants were first organized into groups comprising 3-6 individuals, determined by the outcomes of the social network algorithm with data received at baseline. The social network algorithm was employed to group each at-risk adolescent (those indicating a higher intention to use tobacco or currently using tobacco) with two-to-five of their closest peers exhibiting a lower intention to use tobacco. Within their groups, participants were instructed to engage in ASPIRE activities followed by game-based social activities within a board game. The duration of board game play varied for each session depending on the length of the assigned ASPIRE activity.

**References for Appendix 1**

1. Khalil GE, McLean D, Ramirez E, Mihaj PP, Zhao B, Dhar B, Khan M. Developing a text-message library for tobacco prevention among adolescents: A qualitative study. Plos one. 2024 Jan 4;19(1):e0296503.
2. Kim Y, Kang S, Vongjaturapat N. Application of transtheoretical model to explain adolescents’ smoking behavior. Journal of Substance Use. 2023 Mar 4;28(2):200-5.
3. Brown EC and Sumichrast RT Evaluating Performance Advantages of Grouping Genetic Algorithms. Engineering Applications of Artificial Intelligence, 2005.18(1):1-12.
4. Krouska A and Virvou M An Enhanced Genetic Algorithm for Heterogeneous Group Formation Based on Multi-Characteristics in Social-Networking-Based Learning. IEEE Transactions on Learning Technologies, 2019.13(3):465-476.
5. Prokhorov AV, Kelder SH, Shegog R, et al. Project Aspire: An Interactive, Multimedia Smoking Prevention and Cessation Curriculum for Culturally Diverse High School Students. Substance use & misuse, 2010.45(6):983-1006.
6. Khalil GE, Kim J, McLean D, Ramirez E, Zhao B, Salloum RG. Identifying adolescents’ gaming preferences for a tobacco prevention social game: A qualitative study. Plos one. 2023 Jul 28;18(7):e0289319.
7. McCarthy M and Southeastern N Experiential Learning Theory: From Theory to Practice. Journal of Business & Economics Research (JBER), 2010.8
8. Bandura A Social Learning Theory General Learning Press. New York, 1977.
