# Supplementary material for: Risk Perception and Knowledge Following a Social Game–Based Tobacco Prevention Program for Adolescents: Pilot Randomized Comparative Trial
Source: JMIR Serious Games. 2024 Nov 5;12:e63296. doi: 10.2196/63296 (PMC11576604; doi:10.2196/63296)
Supplement: Multimedia Appendix 3 [file games_v12i1e63296_app3.docx]

**Multimedia Appendix 3.** Main study measures

| Measures (α^a^) | Description | |
| --- | --- | --- |
| **Tobacco-related Measures** | | |
| **Ever use of vaping products; Ever use of cigarettes; Ever use of cigars or little cigars** | Using the Minnesota smoking index (MSI), we measured the status of vaping, smoking cigarettes, and smoking cigars or little cigars. We presented respondents with a figure and description of each product and asked them to pick a choice between never having used the product, not even in part, to using the product for more than five sessions. Considering that a vaping product can be continuously used, a session for vaping was defined as one minute straight of vaping [29]. “Ever use” was assigned automatically to those who picked any choice but “never use”. | |
| **Perceived risk of vaping (0.88); Perceived risk of conventional tobacco use (0.96)** | Five items for each outcome: The first item on a 4-point Likert scale, from no risk to great risk, asks respondents how much they think people risk harming themselves if they use each of the tobacco products (vaping products, cigarettes, cigars, and little cigars). The four remaining items on a 4-point Likert scale, from strongly disagree to strongly agree, presenting statements concerning the likelihood of these products contributing to medical issues like reproductive problems, respiratory ailments, or heart disease [30,31]. Perceived risk of vaping is the average score on these items for vaping products, and perceived risk of conventional tobacco use is the average score on these items for cigarettes, cigars, and little cigars. | |
| **Tobacco Knowledge (0.68)** | We developed a list of 22 questions with multiple-choice and true-or-false answers, testing respondents on their knowledge regarding tobacco in general, vaping products, and conventional products. The questions were based on the health messages within Storm-Heroes and ASPIRE. | |
| **Manipulation Check Measures** | | |
| **Perceived social interactivity (0.98)** | Measured with 17 items from Coursaris and Sung (2012), such as ”The program seemed to facilitate communication between people” and “The program allowed for conversation [32]” | |
| **Attitude toward the program (0.95)** | We used an 11-point semantic differential scale for attitude with four items (eg, dislike/like and not well designed/very well designed) [33]. | |
| **Visual aesthetics (0.97)** | We measured the aesthetic attributes of the program (eg, enjoyment of the colors and graphics) [34,35]. With a set of four items, answer choices ranged from 0=not at all to 10=very much. | |
| **Emotional involvement** | We used a single item asking participants how much they felt emotionally involved in the program [36]. Answer choices ranged from 0=not at all to 10=very much. | |
| **Attention Measures** | | |
| **General attention** | To differentiate between attention and distraction, we presented respondents with a single statement: “I paid attention to the program more than to what was happening around me”. Answer choices ranged from 0=not at all to 10=very much. | |
| **Distraction** | We presented respondents with a single statement: “When I was going through the program, I was distracted by activity around me”. Answer choices ranged from 0=not at all to 10=very much. | |
| **Recognition of program images** | We presented participants with a set of images from ASPIRE, images from Storm-Heroes, and images irrelevant to the programs. With a single question, we asked respondents which images they recall from the program. This method has been commonly used in health media campaigns [37-39]. | |
| **Personal Experience with The Program** | | |
| **Perceived usability (0.92)** | Perceived usability was measured using three items from the usability-playability dimension of the game user experience satisfaction scale (GUESS) [34,35]. Examples of statements included: “it was easy to learn how to play the game” and “I found the material of the game to be straightforward”. Answer choices ranged from 0=not at all to 10=very much. | |
| **Level of fun** | We used a single item, with an 11-point semantic differential scale ranging from “not at all fun” to “fun” [33]. Answer choices ranged from 0=not at all to 10=very much. | |
| **Narrative quality (0.97)** | This measure included three items from the narrative dimension of GUESS (eg, “The story was well developed” and “I could identify with the characters”) [34,35]. Answer choices ranged from 0=not at all to 10=very much. | - |
| **Program enjoyment (0.98)** | This measure included three items related to enjoyment and entertainment value of the program, such as “I enjoyed the program” [26]. Answer choices ranged from 0=not at all to 10=very much. | - |
| **Creative freedom (0.91)** | This measure included three items related to the freedom of being creative during the program [34,35], such as “I felt creative during the program” and “The program gave me enough freedom to act how I want”. Answer choices ranged from 0=not at all to 10=very much. | - |
| **Communication Measures** | | |
| **Engagement in discussions about tobacco or the program** | With “yes” and “no” as answer choices, we asked respondents if they talked to other people during the program. If the answer was “yes”, respondents were then asked an open-ended question: “In a few words, what is the topic that stood out the most in your conversations?” Engagement in the discussion was coded 1 if respondents reported talking about the program or tobacco in a positive light. It was coded 0 otherwise. |  |

^a^Reliability coefficients with Cronbach’s α were calculated when applicable, from baseline data except for measures with data collected at post-test only.
