# Supplementary material for: Risk Perception and Knowledge Following a Social Game–Based Tobacco Prevention Program for Adolescents: Pilot Randomized Comparative Trial
Source: JMIR Serious Games. 2024 Nov 5;12:e63296. doi: 10.2196/63296 (PMC11576604; doi:10.2196/63296)
Supplement: Multimedia Appendix 4 [file games_v12i1e63296_app4.docx]

**Multimedia Appendix 3: Participant characteristics based on attrition**

| Characteristics | Total Sample  (n = 74) | Continued to 1.5-month follow-up | Did not continue to follow-up | *P* ^a^ |
| --- | --- | --- | --- | --- |
| **Age, n (%)** |  |  |  |  |
| 13 years or under | 40 (55.56%) | 24 (55.81%) | 16 (55.17%) | 0.957 |
| Over 13 years | 32 (44.44%) | 19 (44.19%) | 13 (44.83%) |  |
| **Sex at birth, n (%)** |  |  |  |  |
| Male | 31 (41.89%) | 19 (45.24%) | 12 (37.50%) | 0.504 |
| Female | 43 (58.11%) | 23 (54.76%) | 20 (62.50%) |  |
| **Race** |  |  |  |  |
| Being Black or African American | 56 (77.78%) | 36 (83.72%) | 20 (68.96%) | 0.140 |
| Not Black or African American | 16 (22.22%) | 7 (16.28%) | 9 (31.03%) |  |
| **Ethnicity, n (%)** |  |  |  |  |
| Being Hispanic/Latino | 16 (22.22%) | 9 (20.93%) | 7 (24.14%) | 0.748 |
| Not being Hispanic/Latino | 56 (77.78%) | 34 (79.07%) | 22 (75.86%) |  |
| **Grades at school, n (%)** |  |  |  |  |
| Mostly A | 35 (47.95%) | 19 (44.19%) | 16 (53.33%) | 0.441 |
| Mostly B or C | 38 (52.05%) | 24 (55.81%) | 14 (46.66%) |  |
| **Parents’ Level of Education** |  |  |  |  |
| Received a college degree | 53 (72.60%) | 33 (76.74%) | 20 (66.66%) | 0.342 |
| Did not receive a college degree | 20 (27.40%) | 10 (23.25%) | 10 (33.33%) |  |
| **Number of detentions at school** |  |  |  |  |
| None | 58 (78.38%) | 31 (72.09%) | 27 (87.10%) | 0.264 |
| One time | 10 (13.51%) | 8 (18.60%) | 2 (6.45%) |  |
| Two or more | 6 (8.11) | 4 (9.30%) | 2 (6.45%) |  |
|  | Values in M (SD) | | | *P* ^b^ |
| **Perceived board game skills** | 3.45 (0.91) | 3.52 (0.74) | 3.35 (1.12) | 0.453 |
| **Number of friends who vape** | 3.14 (12.32) | 2.21 (4.28) | 4.53 (18.88) | 0.444 |
| **Number of friends who smoke** | 0.68 (2.54) | 1.09 (3.21) | 0.04 (0.19) | 0.092 |
| **Perceived risk of vaping** | 3.15 (0.95) | 3.33 (0.77) | 2.90 (1.13) | 0.057 |
| **Perceived risk of conventional tobacco use** | 3.31 (0.82) | 3.43 (0.67) | 3.13 (0.99) | 0.123 |
| **Tobacco Knowledge** | 10.30 (3.07) | 10.74 (3.17) | 9.45 (2.76) | 0.127 |

^a^Significance testing with χ^2^ test (categorical variables)

^b^Significance testing with ANOVA (continuous variables)
